# Antimicrobial and Antibiofilm Effect of Brazilian Green Propolis Aqueous Extract against Dental Anaerobic Bacteria

**DOI:** 10.3390/molecules27238128

**Published:** 2022-11-22

**Authors:** Maria Angélica de Sá Assis, Lucas de Paula Ramos, Amjad Abu Hasna, Talita Suelen de Queiroz, Thaís Cristine Pereira, Patrícia Michelle Nagai de Lima, Andresa Aparecida Berretta, Maria Cristina Marcucci, Cláudio Antonio Talge Carvalho, Luciane Dias de Oliveira

**Affiliations:** 1 Department of Biosciences and Oral Diagnosis, Institute of Science and Technology, São Paulo State University (ICT-UNESP), São José dos Campos 12245-000, Brazil; 2 Department of Restorative Dentistry, Endodontics Division, Institute of Science and Technology, São Paulo State University (ICT-UNESP), São José dos Campos 12245-000, Brazil; 3 Research and Development Laboratory, Apis Flora Industrial e Comercial Ltda., Rua Triunfo, 945, Ribeirão Preto 14020-670, Brazil

**Keywords:** green propolis, antimicrobial action, anaerobic bacteria

## Abstract

Green propolis may represent a promising therapeutic alternative against dental anaerobic pathogens because of its antimicrobial action. This study aimed to evaluate the antimicrobial and antibiofilm actions of Brazilian green propolis aqueous extract (BGP-AqExt) against dental anaerobic bacteria. The minimum inhibitory concentration (MIC) and minimum microbicide concentration (MMC) of the extract were determined against the standard strains (ATCC) of *Fusobacterium nucleatum*, *Parvimonas micra*, *Prevotella intermedia, Porphyromonas gingivalis* and *Porphyromonas endodontalis*. BGP-AqExt was chemically characterized by high-performance liquid chromatography with diode-array detection (HPLC-DAD) analysis. Antibiofilm action was measured by MTT and crystal violet tests. The data were statistically analyzed by ANOVA and Tukey (5%) tests. The extract had antimicrobial action against all tested anaerobic bacteria, with an MIC value of 55 mg/mL for all bacteria, an MMC of 27.5 mg/mL for *F. nucleatum* and *P. micra* and 55 mg/mL for *P. intermedia*. Chemically, BGP-AqExt is composed of quercetin, gallic acid, caffeic and p-coumaric acid, drupani, kaempferol and Artepillin C. Significant reductions in biomass and metabolic action of biofilms were found after BGP-AqExt application. Therefore, BGP-AqExt has an antimicrobial and antibiofilm effect against dental anaerobic bacteria.

## 1. Introduction

The oral cavity is subjected to endodontic and periodontal infections, among others [1,2,3], in which diverse dental anaerobic microorganisms are involved in these infections, including *Porphyromonas gingivalis, Fusobacterium nucleatum, Parvimonas micra, Porphyromonas endodontalis* and *Prevotella intermedia* [4,5,6]. Conventionally, sodium hypochlorite, chlorhexidine and calcium hydroxide are used as antimicrobial agents to combat the microorganisms that cause such infections [1,7,8,9]; however, there is a continuous need for research for more effective and less toxic natural compounds [10]. 

Additionally, there is an increased need for new antibiotics and antimicrobial agents to treat systemic infections [11] because of the irresponsible consumption and overuse of conventional antibiotics [12]. The emergence of new multidrug-resistant microorganisms is related to the uncontrolled use of antibiotics, making the disinfection of these microorganisms a challenge that reinforces the need for natural antimicrobial products [13,14,15].

Phytotherapy is making great efforts in this aspect, in which diverse medicinal plants were presented recently as alternative antimicrobials to treat diverse infections because of their antimicrobial action and biocompatibility [16,17,18,19]. Propolis is a resinous substance enriched by biologically active molecules and is produced by *Apis mellifera* through the collection of different plant parts [20]. This complex substance has numerous proprieties such as antibacterial, antifungal and immunomodulatory action [21]. Its antimicrobial mechanism is achieved by reducing the ATP synthesis, decreasing the microbial mobility, increasing th cell membrane permeability, disturbing the cell membrane potential and inducing the host immune response [22]. 

The chemical composition of propolis varies according to the bees’ geographic location and habitat conditions, such as food and lighting [23]. There are different types of propolis, in accordance with the botanic origin and bee species, including green, brown, red and yellow propolis [20] originating from different geographic regions [24]. The Brazilian green propolis botanic source is *Baccharis dracunculifolia’s* DC, popularly known as ‘wild rosemary’, a plant from the *Asteraceae* family [20]. The green propolis from Taiwan and Brazil was tested against *Listeria monocytogenes, Ruminococcus albus, Ruminococcus flavefaciens, Staphylococcus aureus* and was found bacteriostatic with a potent antimicrobial action [25,26].

To the best of our knowledge, the Brazilian green propolis antibiofilm action was not tested against some dental anaerobic bacteria, including *P. gingivalis, F. nucleatum, P. micra, P. endodontalis* and *P. intermedia*. Therefore, the aim of this study was to evaluate the antimicrobial and antibiofilm action of the Brazilian green propolis aqueous extract (BGP-AqExt) against these bacteria. The null hypothesis was that the Brazilian green propolis has no antibiofilm action against dental anaerobic bacteria.

## 2. Results

### 2.1. BGP-AqExt Characterization

**Figure 1:**
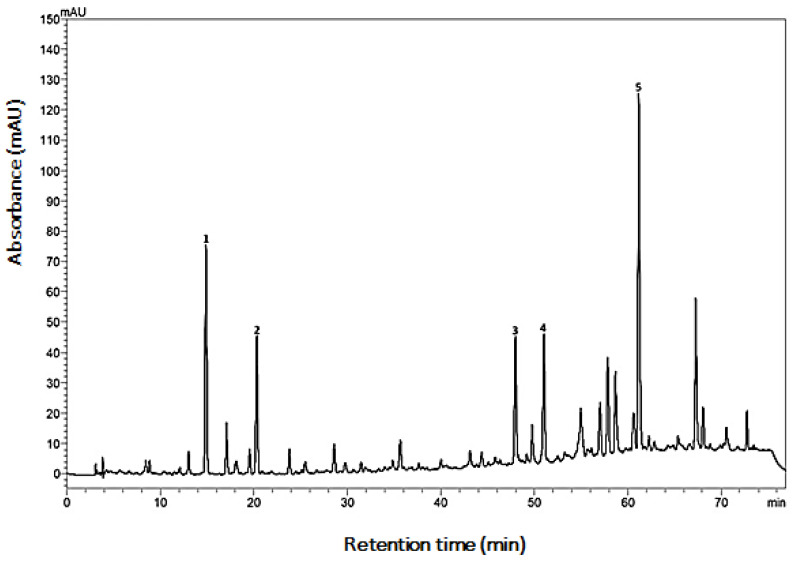
Chemical fingerprint obtained using C18-RP column for BGP-ExtAq. Peak 1 = caffeic acid, peak 2 = p-coumaric acid, peak 3 = drupanin, peak 4 = kaempferide and peak 5 = Artepillin C.

The characterization of BGP-AqExt showed that it has the characteristic chemical fingerprint of green propolis, obtained from the *Baccharis dracunculifolia* botanical source (Figure 1).

The extract was composed of 1.178 ± 0.006 mg/g of caffeic acid, 0.451 ± 0.001 mg/g of p-coumaric acid, 0.680 ± 0.004 mg/g of kaempferide (expressed in kaempferol), 0.899 ± 0.004 mg/g of drupanin and 3.773 ± 0.079 mg/g of Artepillin C as shown in Table 1. 

### 2.2. Antibacterial Action of BGP-AqExt on Planktonic Strains

The minimum inhibitory concentration (MIC) value of the BGP-AqExt was 55 mg/mL for all the tested microorganisms. However, the minimum microbicidal concentration (MMC) value was 27.5 mg/mL for *F. nucleatum* and *P. micra* and 55 mg/mL for *P. intermedia*. Conversely, it was not possible to determine the MMC value of BGP-AqExt for *P. gingivalis* and *P. endodontalis* (Table 2).

### 2.3. Crystal Violet Assay

**Figure 2:**
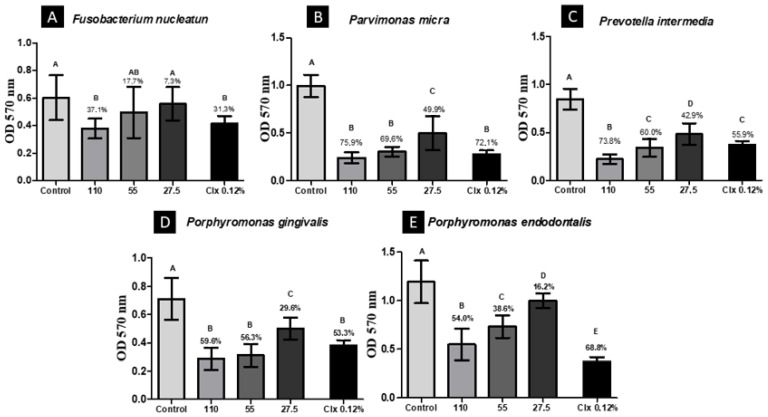
Biomass reduction after contact for 5 min with the BGP-AqExt. Legend: OD—Optical Density; Control: BHI broth; Clx—Chlorhexidine; Values expressed in mg/mL; Different uppercase letters (A–E) indicate statistically significant difference (ANOVA, Tukey, *p* < 0.05).

BGP-AqExt (110 mg/mL) was the most effective concentration in which the biomass was reduced to 37.1, 75.9, 73.8, 59.6 and 54.0% of *F. nucleatum, P. micra, P. intermedia, P. gingivalis* and *P. endodontalis*, respectively, after of contact for five minutes. This reduction has a statistically significant difference when compared to the saline solution group. The antibiofilm action of BGP-AqExt (110 mg/mL) was more effective than chlorhexidine against *P. intermedia* and as effective against *F. nucleatum, P. micra* and *P. gingivalis* (Figure 2).

### 2.4. Analysis of Cell Viability of Microorganisms by MTT

**Figure 3:**
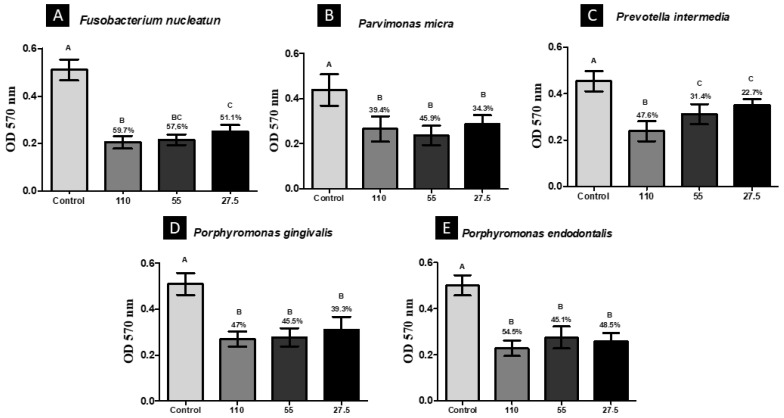
Viability reduction after contact for 5 min with the BGP-AqExt. Legend: OD—Optical Density; Control: BHI broth; Values expressed in mg/mL; Different uppercase letters (**A**–**E**) indicate statistically significant difference (ANOVA, Tukey, *p* < 0.05).

Again, BGP-AqExt (110 mg/mL) was the most effective concentration in which the biomass was reduced to 59.7, 47.6, 47.0 and 54.5% of *F. nucleatum, P. intermedia, P. gingivalis* and *P. endodontalis*, respectively, after contact for 5 min, except against *P. micra*, in which BGP-AqExt (55 mg/mL) was more effective than BGP-AqExt (110 mg/mL). BGP-AqExt (110 mg/mL) antibiofilm action has a statistically significant difference when compared to the saline solution group (Figure 3).

## 3. Discussion

There is no doubt about the efficacy of chlorohexidine as an antimicrobial agent [27,28]; thus, it was used in this study as a positive control group. In this study, the antibiofilm action of BGP-AqExt (110 mg/mL) was more effective than chlorhexidine against *P. intermedia* and as effective as chlorhexidine against *F. nucleatum, P. micra* and *P. gingivalis*. Therefore, the null hypothesis of this study was rejected. 

In the literature, the BGP-AqExt was tested against multidrug-resistant aerobic strains of *Klebsiella pneumoniae* and *Pseudomonas aeruginosa*, in which the extract was effective and presented a rapid onset against the tested microorganisms [29]. Similarly, in the present study, it was effective against all the tested anaerobic bacteria. BGP-AqExt showed bactericidal action, with MIC 55 mg/mL for all the tested microorganisms and MMC value of 27.5 mg/mL for *F. nucleatum* and *P. micra* and 55 mg/mL for *P. intermedia*.

In another study, the antimicrobial action of Pakistani propolis was tested against 35 clinical isolates of pigmented anaerobic periodontal pathogens, namely *Porphyromonas asaccharolytica*, *P. gingivalis*, *P. intermedia* and *Prevotella melaninogenica*, by means of an agar diffusion assay, and it was observed that all the strains were significantly sensitive to the ethanol extract of propolis [30]. These results indicate that this propolis has a potent antimicrobial action against such anaerobic bacteria, corroborating our study carried out with Brazilian green propolis, which evaluated the antimicrobial action against anaerobic strains but using the microdilution test in broth. Furthermore, ethanol extracts of propolis, collected from four different regions of Turkey and Brazil, had their antimicrobial action analyzed against nine anaerobic strains. All strains were susceptible, and MIC values ranged from 4 to 512 μg/mL for propolis action [31]. 

The reduction of *P. gingivalis* biofilm using a honey-propolis (Manuka honey) compound was evaluated after contact for 24 h with the compound; it reduced the concentration of viable bacteria when compared to controls without the addition of honey and propolis [32], proving the antibiofilm action of propolis against *P. gingivalis*, as found in the present study. Furthermore, the ethanol extract of propolis from southern Brazil was evaluated against different endodontic bacteria, including *Prevotella nigrescens*, *F. nucleatum*, *Actinomyces israelii*, *Clostridium perfringens* and *E. faecalis* by macrodilution. As a result, *P. nigrescens* was the most susceptible, *F. nucleatum* and *C. perfringens* had intermediate results and *A. israelii* and *E. faecalis* were the most resistant [33]. Similarly, in the present study, an intermediate reduction was observed in the biomass of the pathogen *F. nucleatum* when submitted to contact with BGP-AqExt.

More recently, the Brazilian red propolis was tested against *P. melaninogenica, Actinomyces viscosus, Prevotella nigrescens, P. endodontalis*, *V. parvula*, *P. gingivalis*, *P. intermedia*, *P. micra* and *F. nucleatum*, in which the red propolis was effective against these pathogens involved in primary endodontic infections, the study indicated the use of the red propolis as endodontic irrigant or intracanal medication in a concentration ranging from 6.25 to 200 mg/mL [34]. Differently, in the present study, lower concentrations of the BGP-AqExt were effective against the same pathogens. 

BGP-AqExt characterization showed that it has the characteristic chemical fingerprint of green propolis, obtained from a *Baccharis dracunculifolia* botanical source, as reported by an anterior study [35]. Caffeic and p-coumaric acid, kaempferide, drupanin and Artepillin C have been found. Despite being aqueous, the BGP-AqExt (Propomax- Apis flora, Brazil) has a high number of flavonoids and total phenols due to the differentiated extraction protocol [29]. The presence of total flavonoids and phenols may explain the antimicrobial effect of the BGP-AqExt [36], as the flavonoids’ antimicrobial mechanism makes it effective against a wide range of microorganisms, including dental ones [37].

Another compound identified was caffeic acid, which has several pharmacological effects, including anticariogenic potential [38]. Artepillin C (3,5-diprenyl-p-coumaric acid), which was also identified, is one of the phenolic compounds (prenyl derivative of p-coumaric acid) present in propolis [22]. This compound has bacteriostatic action with membrane bubbles against *P. gingivalis*, agreeing with our study that did not find the value of MMC in this species, considering it as bacteriostatic.

The antibiofilm of BGP-AqExt proved in the present study of five dental anaerobic bacteria, encourages its use as an endodontic irrigant, intracanal medication, mouthwash, toothpaste component, or as a medicament for clinical use to combat endodontic, periodontal and oral cavity infections.

## 4. Materials and Methods

### 4.1. Brazilian Green Propolis (BGP) Aqueous Extract (AqExt) Characterization

The use of plant parts in the present study complies with international, national and/or institutional guidelines. BGP-AqExt (110 mg/mL of propolis—Propomax, Batch: 0030 015 17) from Apis Flora (Ribeirão Preto, Brazil) was used in antimicrobial and antibiofilm tests. BGP-AqExt was obtained from a blend of Brazilian green raw propolis types. This blend was frozen and triturated to be extracted by maceration and percolation with ethanol and purified water solution 70:30% *w*/*w*. The extract obtained suffered a concentration process to eliminate the ethanol content, according to a previously published study [39]. The BGP-AqExt obtained was chemically characterized according to the available methodology for propolis extract evaluation, as will be described below. The used propolis raw material was obtained from *Apis mellifera* Africanized bees, a poly-hybrid obtained from both exogenous species (Europe and Africa) introduced to Brazil; thus, according to the Brazilian Regulations, this material is dispensed of CGEN approval (Genetic Heritage Management Council). 

Total phenolics and flavonoids were determined using solvents of analytical purity. Phosphoric acid, sodium carbonate and methanol supplied by Synth^®^ (São Paulo, Brazil); aluminum chloride (Cinética^®^, Brazil), sodium tugstate (Química Moderna^®^, Mogi das Cruzes, Brazil), phosphomolybdic acid (Nuclear^®^) and purified water (Milli-Q^®^, Darmstadt, Germany) was employed. The chemical reference substances used for this evaluation were gallic acid and quercetin, supplied by ChormaDex (Irvine, AB, Canada). The testing was performed using spectrophotometer (Shimadzu, Barueri, Brazil), analytical balance (Mettler Toledo, Columbus, OH, USA), automatic micropipettes (LabMate, St Albans, UK), centrifuge and ultrasound bath. BGP-AqExt was characterized according to total flavonoid content using quercetin as chemical reference standard with colorimetric method using aluminum chloride as reagent and 425 nm wavelength [29,40]. Total phenolic content was quantified using gallic acid as chemical reference standard using Folin–Denis colorimetric method with some adjustments [29,39]. For the high-performance liquid chromatography (HPLC) analysis, proper HPLC purity solvents were used, such as methanol, ultrapure water (resistivity 18 MΩ) and formic acid.

High-performance liquid chromatography with diode-array detection (HPLC-DAD) analysis was performed according to previously developed and validated method for propolis water extract [39], using C18 reverse column, with the chemical references’ substances caffeic acid, p-coumaric acid, kaempferol (Sigma-Aldrich, St. Louis, MO, USA), drupanin and Artepillin C, which were isolated and identified by Prof. Dr. Jairo Kenupp Bastos (Faculty of Pharmaceutical Sciences of Ribeirão Preto, University of São Paulo–FCFRP/USP) and kindly donated by him.

### 4.2. Bacterial Strain Isolation 

Five different strains of bacteria were reactivated in enriched in Brucella broth (BD—Heidelberg, Germany) with 1% hemin and 1% vitamin K (menadione) (Sigma-Aldrich, St. Louis, MO, USA), then were seeded in enriched Brucella agar at 37 °C for 48 h in anaerobic chamber Whitley DG250 Workstation (Don Whitley Scientific Limited, Shipley, West Yorkshire, UK).

The susceptibility of *F. nucleatum* (ATCC 25586), *P. micra* (ATCC 23195), *P. endodontalis* (ATCC 35406), *P. gingivalis* (ATCC 33277) and *P. intermedia* (ATCC 33563) was determined in planktonic culture by broth microdilution assay accordingly to Clinical and Laboratory Standards Institute (CLSI), standard M11-A7 (2012), to determine the minimum inhibitory concentration (MIC) and minimum microbicide concentration (MMC). 

### 4.3. Bacterial Inoculum Preparation

Bacterial inocula were prepared in sterilized saline solution (NaCl 0.9%) and standardized at (1 × 10^8^ CFU/mL) according to the MacFarland scale in a spectrophotometer (Micronal, São Paulo, Brazil). Then, the extract was diluted (1:2) in 100 µL Brucella broth (Himedia, Mumbai, Índia) in microtiter plate wells (TPP, Switzerland). Later, 100 µL of standardized inoculum was added to each well. After incubation (48 h/37 °C), MIC values were determined in the first well with absence of microbial turbidity, next to the well with apparent microbial growth. MMC values were determined, and 10 µL of MIC and a concentration above and below MIC were seeded in Brucella agar. After incubation (48 h/37 °C), MMC corresponded to the well with no grown bacteria and with the lowest concentration of BGP-AqExt [4,41].

### 4.4. Antibiofilm Action

After biofilm formation, as detailed in anterior study [29], the supernatant was discarded, and the biofilms were treated with BGP-AqExt for 5 min at concentrations of 110, 55 and 27.5 mg/mL. Sterilized NaCl 0.9% and chlorohexidine 0.12 mL were used as control groups. A total of 12 replicates were performed per experimental group. Finally, to remove the affected bacterial cells, the wells were washed with sterilized NaCl 0.9%. Posteriorly, the biofilm mass and metabolic activity were evaluated by crystal violet and MTT assays, respectively. 

### 4.5. Crystal Violet (CV) Assay

The treated bacteria were fixed with 200 µL/well of methanol for 20 min. Then, it was removed, and the microplates were incubated for 24 h at 37 °C. After incubation, 200 µL/well of 1% (*v*/*v*) CV solution was added for 5 min. Subsequently, CV solution was discarded, and the wells were washed with sterilized NaCl 0.9%. After, 33% (*v*/*v*) acetic acid was added, and the plate was subjected to agitation for 10 min. Finally, a spectrophotometer (Bio-Tek Instruments, Winooski, GU, USA) was used to measure the absorbance of the wells at 570 nm, and data generated were converted to cell viability percentage [4].

### 4.6. MTT Assay

The 3-(4,5-Dimethyl-2-thiazolyl)-2,5-diphenyl-2H-tetrazolium bromide (MTT) (Sigma-Aldrich, Saint Louis, MO, USA) was suspended in phosphate-buffered saline (PBS) at 0.5 mg/mL. MTT solution (100 µL/well) was added to microtiter plate wells. After 1-h incubation at 37 °C, covered from light, the supernatant was discarded, and 100 µL/well of dimethyl sulfoxide (DMSO) was added. Immediately after, the microplates were incubated for 10 min at 37 °C and then were agitated in vortex for additional 10 min at room temperature. Finally, a spectrophotometer (Bio-Tek Instruments, Winooski, VT, USA) was used to measure the absorbance of the wells at 570 nm, and data generated were converted to cell viability percentage [41].

### 4.7. Statistical Analysis

Data were submitted to a normality test and then were analyzed by one-way ANOVA and Tukey test considering significance level α ≤ 0.05 using GraphPad Prism 6 (La Jolla, CA, USA).

## 5. Conclusions

The Brazilian green propolis aqueous extract at 110 mg/mL has effective antibiofilm action against *P. gingivalis, F. nucleatum, P. micra, P. endodontalis* and *P. intermedia* after contact for five minutes, encouraging its use as an endodontic irrigant, intracanal medication, mouthwash, toothpaste component or as a medicament for clinical use to combat endodontic, periodontal and oral cavity infections. Further studies in animals and clinical studies should be carried out to evaluate the antibiofilm action of Brazilian green propolis. 

## Figures and Tables

**Table 1:** Chemical characterization of BGP-AqExt (Propomax—0030 015 17) according to colorimetric and High-performance liquid chromatography (HPLC) methods.

Parameters/Samples	Mean ± Standard Deviation (mg/g)
Caffeic acid	1.178 ± 0.006
p-Coumaric acid	0.451 ± 0.001
Drupanin	0.899 ± 0.004
Kaempferide (as kaempferol)	0.680 ± 0.004
Artepillin C	3.773 ± 0.079
Total Flavonoids as quercetin	5.816 ± 0.110
Total Phenol Content as gallic acid	17.026 ± 0.109

**Table 2:** MIC and MMC values of BGP-AqExt against the tested anaerobic microorganisms.

Microorganism	BGP-AqExt (mg/mL)	
	MIC	MMC
*F. nucleatum*	55	27.5
*P. micra*	55	27.5
*P. intermedia*	55	55
*P. gingivalis*	55	abs
*P. endodontalis*	55	abs

Legend: BGP-AqExt—Brazilian green propolis aqueous extract; Abs—Absent; MIC—Minimum Inhibitory Concentration; MMC—Minimum Microbicidal Concentration.

## Data Availability

The data used to support the findings of this study are available upon request with the corresponding author d.d.s.amjad@gmail.com.

## Sample Availability

The samples of this study are available upon request with the corresponding author d.d.s.amjad@gmail.com.
